# Effects of Metal Nanoparticles on Methane Production from Waste-Activated Sludge and Microorganism Community Shift in Anaerobic Granular Sludge

**DOI:** 10.1038/srep25857

**Published:** 2016-05-11

**Authors:** Tao Wang, Dong Zhang, Lingling Dai, Yinguang Chen, Xiaohu Dai

**Affiliations:** 1 State key laboratory of pollution control and Resources reuse, School of Environmental Science and Engineering, Tongji University, 1239 Siping Road, Shanghai 200092, China

## Abstract

Extensive use of nanoparticles (NPs) in consumer and industrial products has led to concerns about their potential environmental impacts; however, the influences of different NPs (e.g., nZVI (nano zero-valent iron), Ag NPs, Fe_2_O_3_ NPs and MgO NPs) on the anaerobic digestion of sludge have not yet been studied in depth. Additionally, a new guideline or the use of different NPs in the anaerobic digestion of sludge should be established to improve the anaerobic digestion of sludge and avoid inhibitory effects. This study investigated the effects of four representative NPs (i.e., nZVI, Ag NPs, Fe_2_O_3_ NPs and MgO NPs) on methane production during the anaerobic digestion of waste activated sludge (WAS). The presence of 10 mg/g total suspended solids (TSS) nZVI and 100 mg/g TSS Fe_2_O_3_ NPs increased methane production to 120% and 117% of the control, respectively, whereas 500 mg/g TSS Ag NPs and 500 mg/g TSS MgO NPs generated lower levels of methane production (73.52% and 1.08% that of the control, respectively). These results showed that low concentrations of nZVI and Fe_2_O_3_ NPs promoted the amount of microbes (Bacteria and Archaea) and activities of key enzymes but that higher concentrations of Ag NPs and MgO NPs inhibited them.

Nanoparticles (NPs) with at least one dimension on the order of 100 nm or less^1^ have commonly been applied in commercial industrial and consumer products, such as antimicrobials, paints, coatings, medicines, foods, cosmetics, catalysts and environmental processes^2^,^3^,^4^, due to their unique physicochemical characteristics (e.g., size, specific surface area, surface structure, solubility and catalytic properties). Although the current predicted environmental dosage of NPs (e.g., Ag NPs, TiO_2_ NPs and ZnO NPs) is at the μg/L or mg/L level, their release into the environment may continuously increase during large-scale production^5^,^6^. The increasing utilization of NP-containing products leads to release of NPs into wastewater treatment plants (WWTPs)^7^,^8^, where the NPs are adsorbed, aggregated and settled via activated sludge^9^,^10^. During this process, large amounts of waste-activated sludge (WAS) are produced; thus, most of the NPs will enter the sludge treatment system. Biological treatment of wastes, such as wastewater and WAS, under anaerobic conditions is a highly sustainable waste treatment process because this technique can reduce environmental pollution with simultaneous recovery of energy (i.e., methane). Many researchers have investigated the behavior of NPs in the environment and their systematic relevant risk^11^,^12^,^13^. However, it remains necessary to study the influences of different NPs (e.g., nZVI (nano zero-valent iron), Ag NPs, Fe_2_O_3_ NPs and MgO NPs) on the anaerobic digestion of sludge.

Studies have demonstrated that many NPs could induce strong inhibitory effects in biological treatment processes. For example, NPs composed of ZnO, CuO, CoO, Mn_2_O_3_, Co_3_O_4_, Ni_2_O_3_ and Cr_2_O_3_ were found to exert significant growth inhibitory effects; these effects were found to relate to membrane damage and oxidative stress responses^14^. Choi *et al.* observed that Ag NPs strongly inhibited the respiration of nitrifying organisms, with an inhibition rate of 86 ± 3%^15^. Mu *et al.* reported that higher dosages of ZnO NPs inhibited the steps of sludge hydrolysis, acidification and methanogenesis during an anaerobic digestion process^16^. However, other studies reported that specific NPs could improve biological treatment processes. For example, long-term exposure to 50 mg/L magnetic NPs led to enhanced wastewater nitrogen removal performance^17^. In anaerobic digestion, exposure to Fe NPs effectively reduced H_2_S in biogas and significantly promoted methane production in certain cases^18^. These results indicated that different types of NPs might show dissimilar behaviors in biological treatment processes; however, new guidelines for the use of different NPs (e.g., nZVI, Ag NPs, Fe_2_O_3_ NPs and MgO NPs) in the anaerobic digestion of sludge should be established to improve the anaerobic digestion of sludge and mitigate inhibitory effects.

Anaerobic digestion of sludge can be achieved using complex microbial communities. Therefore, the diversity of microbial populations and the stability of the bacterial community structure play important roles in anaerobic digestion of sludge. Xia *et al.* reported that the toxicity of ZnO NPs to the lysosome and mitochondria of a microbial community was caused by the disruption of intracellular Zn homeostasis^19^. Other researchers found that achieving the optimum concentration of released metallic ions could increase the activities of microbial communities. Li *et al.* indicated that Fe^2+^ released from Fe NPs (nZVI) could combine with S^2−^ during the anaerobic digestion process to relieve the inhibitory effects of S^2−^ on microbial communities^20^. It is difficult to describe the different effects of NPs (e.g., nZVI, Ag NPs, Fe_2_O_3_ NPs and MgO NPs) on the microorganisms in a WAS anaerobic digestion system based on the effects of current NPs because a varity of species of bacteria are present in WAS digestion systems. The impacts of NPs (e.g., nZVI, Ag NPs, Fe_2_O_3_ NPs and MgO NPs) on microorganism communities involved in anaerobic digestion systems have yet to be investigated in the literature.

The objectives of this study are as follows (see also Fig. 1). First, this study aimed to determine the effects of four types of NPs (nZVI, Ag NPs, Fe_2_O_3_ NPs and MgO NPs) on methane production during the anaerobic digestion of sludge. Second, this study aimed to reveal the effects of different NPs on the anaerobic granular sludge surface and metal ions as well as direct interspecies electron transfer (DIET) effects. Finally, this study attempted to explore the shift in the microorganism community in anaerobic granular sludge after exposure to different NPs using fluorescence *in situ* hybridization (FISH) and real-time fluorescent quantitative polymerase chain reaction (FQ-PCR). The activities of certain key enzymes that are associated with the anaerobic digestion process, such as protease, acetate kinase (AK) and coenzyme F_420_, were also considered.

## Results

### Effects of NP Exposure on Methane Production

**Figure 2:**
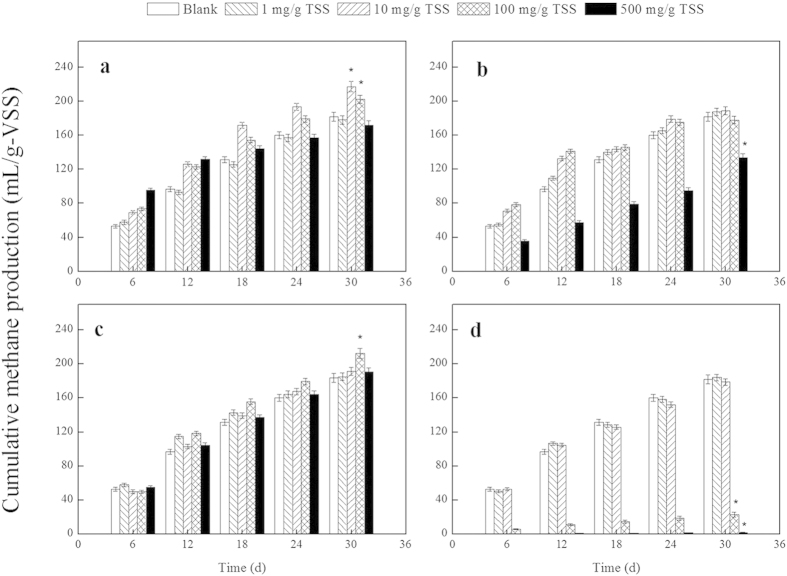
Effects of different dosages of nZVI (a), Ag NPs (b), Fe_2_O_3_ NPs (c) and MgO NPs (d) on methane oduction during WAS anaerobic digestion after different fermentation times. Error bars represent the standard deviations of duplicate tests.

The presence of 0.1 mM sodium dodecylbenzene sulfonate (SDBS), which was added as the dispersing reagent, in the WAS anaerobic digestion experiments had no significant effect on methane production, which is consistent with other studies^21^. The blank test was performed with an SDBS concentration of 0.1 mM in the WAS anaerobic digestion experiments but without NPs. Figure 2 and Table S1 (Supplementary Information) show the effects of different dosages of nZVI, Ag NPs, Fe_2_O_3_ NPs or MgO NPs on methane production during WAS anaerobic digestion at different fermentation times. As shown in Fig. 2a–d, the cumulative methane production levels were 177.92, 187.39, 184.95 and 183.95 mL/g volatile suspended solids (VSS) for the nZVI, Ag NPs, Fe_2_O_3_ NPs and MgO NPs, respectively, each at 1 mg/g TSS. These results were nearly identical to those observed in the blank test (i.e., 181.72 mL/g VSS), which suggests that 1 mg/g total suspended solids (TSS) nZVI, Ag NPs, Fe_2_O_3_ NPs or MgO NPs had no measurable effect on methane generation (*p* > 0.05). However, when the dosages of nZVI and Fe_2_O_3_ NPs were 10 and 100 mg/g TSS, respectively, the cumulative methane production levels were 217.16 and 212.43 mL/g VSS, respectively (i.e., 120% and 117% of the control; *p* < 0.05) (Fig. 2a,c). When the sludge was exposed to 500 mg/g TSS Ag NPs or MgO NPs, the cumulative methane production levels decreased to 133.60 and 1.97 mL/g VSS, respectively (i.e., 73.52% and 1.08% of the control; *p* < 0.05) (Fig. 2b,d). During the 30-day fermentation process, the impacts of nZVI, Ag NPs, Fe_2_O_3_ NPs and MgO NPs on methane production were dependent on the dosages used (i.e., 1, 10, 100 and 500 mg/g TSS). However, lower concentrations of nZVI and Fe_2_O_3_ NPs (i.e., 10 and 100 mg/g TSS) promoted methane production, whereas higher concentrations of Ag NPs and MgO NPs (i.e., 500 mg/g TSS) inhibited methane production. In analyses of the mechanism underlying these effects, tests with 10 mg/g TSS nZVI, 100 mg/g TSS Fe_2_O_3_ NPs, 500 mg/g TSS Ag NPs, and 500 mg/g TSS MgO NPs are indicated by nZVI-10, Fe_2_O_3_ NPs-100, Ag NPs-500, and MgO NPs-500, respectively.

### Effects of NPs on the Anaerobic Granular Sludge Surface, Metal Ion Release and DIET Impact

**Figure 3:**
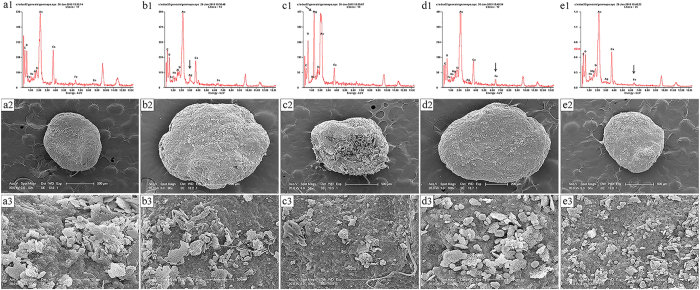
Scanning electron micrograph (SEM) images (a2–e2, a3–e3) and energy-dispersive X-ray (EDX) analysis (a1–e1) of anaerobic granular sludge in the reactors exposed to Ag NPs, MgO NPs, nZVI and Fe_2_O_3_ NPs at the indicated concentrations. Control (**a1–a3**), Ag NPs-500 (**b1–b3**), MgO NPs-500 (**c1–c3**), nZVI-10 (**d1–d3**), Fe_2_O_3_ NPs-100 (**e1–e3**).

As shown in Fig. 3, the anaerobic granular sludge (AGS) surfaces were rougher in the reactors exposed to different concentrations of Ag NPs, MgO NPs, nZVI and Fe_2_O_3_ NPs (nZVI-10, Ag NPs-100, Fe_2_O_3_ NPs-100, Ag NPs-500, and MgO NPs-500) than in the control. The SEM and EDX analyses showed that many NPs were adsorbed onto the AGS surfaces after long-term exposure to the NPs. The combination of NPs and AGS was primarily due to electrostatic interactions and the high specific area of the NPs^1^. The cell membrane integrity of the activated sludge was measured by a Lactate dehydrogenase (LDH) release assay. As shown in Fig. S1 (Supplementary Information), the results of the LDH release assays indicated that the contents of lactate dehydrogenase, which is a cell-membrane damage marker, in the reactors exposed to 500 mg/g TSS Ag NPs or 500 mg/g TSS MgO NPs were considerably higher than those in the reactors exposed to 10 mg/g TSS nZVI or 100 mg/g TSS Fe_2_O_3_ NPs, suggesting that high concentrations of Ag NPs or MgO NPs could damage cell membranes.

**Figure 4:**
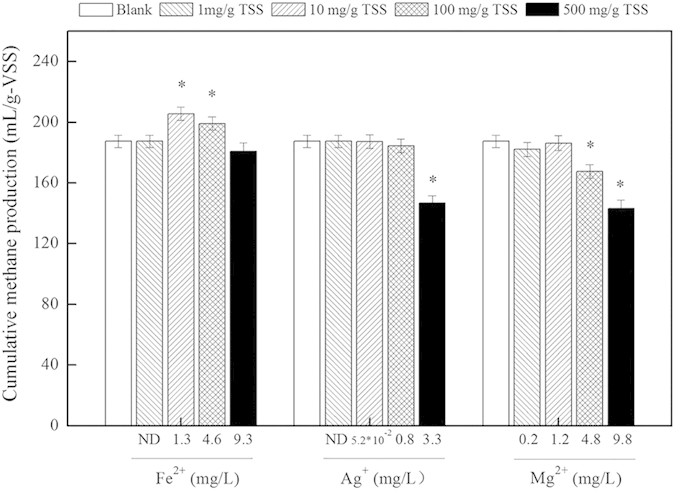
Effects of the Fe^2+^, Ag^+^, and Mg^2+^ released from nZVI, Ag NPs and MgO NPs on methane production during the WAS digestion process. Error bars represent the standard deviations of duplicate tests.

The corresponding released metal ion concentrations for different dosages of Ag NPs, MgO NPs, nZVI and Fe_2_O_3_ NPs are shown in Fig. 4. However, Fe_2_O_3_ NPs do not dissolve easily in their liquid phase under near-neutral conditions. Although no ions were released from Fe_2_O_3_ NPs, this study indicated that exposure to 100 mg/g TSS Fe_2_O_3_ NPs had a positive effect on the anaerobic digestion of sludge. The possible mechanisms underlying this process are explored below. The average concentrations of the Fe^2+^, Ag^+^, and Mg^2+^ released from 10 mg/g TSS nZVI, 500 mg/g TSS Ag NPs and 500 mg/g TSS MgO NPs were 1.3, 3.3 and 9.8 mg/L, respectively (Fig. 4). Batch tests were conducted to examine the potential effects of these dosages of Fe^2+^, Ag^+^ and Mg^2+^ on the anaerobic digestion of sludge. The sludges exposed to 1.3 mg/L Fe^2+^, 3.3 mg/L Ag^+^ and 9.8 mg/L Mg^2+^ showed tendencies similar to those of sludges exposed to 10 mg/g TSS nZVI, 500 mg/g TSS Ag NPs and 500 mg/g TSS MgO NPs. Lower concentrations (i.e., 1.3 and 4.6 mg/L) of Fe^2+^ were found to facilitate the anaerobic digestion of sludge, whereas higher concentrations (i.e., 3.3 and 9.8 mg/L) of Ag^+^ and Mg^2+^ were found to inhibit the anaerobic digestion of sludge. Comparing the anaerobic digestion of sludge induced by nZVI, Ag NPs or MgO NPs with that induced by the corresponding amounts of Fe^2+^, Ag^+^ and Mg^2+^ shows that the released Fe^2+^, Ag^+^ and Mg^2+^ were primarily responsible for the facilitatory and/or inhibitory impacts of nZVI, Ag NPs and MgO NPs.

### Shift in the Microorganism Community of Anaerobic Granular Sludge after Exposure to NPs

**Figure 5:**
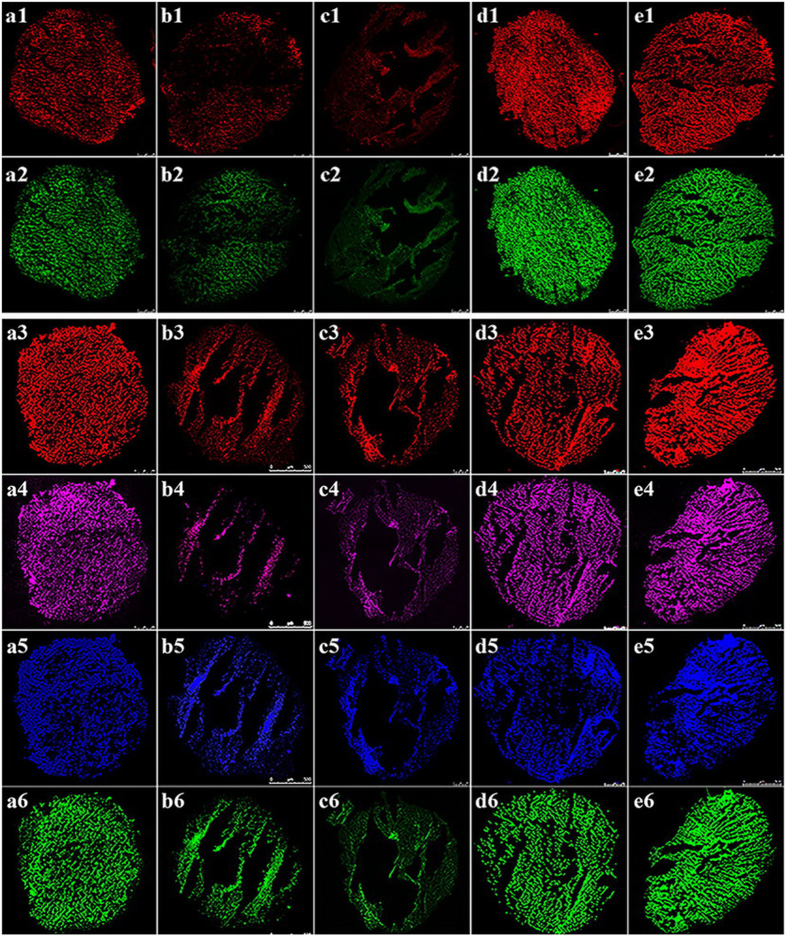
Fluorescence *in situ* hybridization of AGS sections of biomass cultured over the long term in the absence of NPs (a1–a6) and with 500 mg/g TSS Ag NPs (b1–b6), 500 mg/g TSS MgO NPs (c1–c6), 10 mg/g TSS nZVI (d1–d6) or 100 mg/g TSS Fe_2_O_3_ NPs (e1–e6) viewed via CLSM and photographed at higher (i.e., 62×) magnification. The Cy-3-labeled Bacteria-domain probe (EUB338) (red, **a1–e1**) and the FAM-labeled Archaea-domain probe (ARC915) (green, **a2–e2**) were simultaneously hybridized with the sections. A Cy-3-labeled α-Proteobacteria probe (ALF968) (red, **a3–e3**), a Cy-5-labeled β-Proteobacteria probe (BET42a) (rose, **a4–e4**), a HEX-labeled Bacteroidetes probe (CFB719) (blue, **a5–e5**), and a FAM-labeled *Methanosaeta* probe (MX825) (green, **a6–e6**) were simultaneously hybridized with the sections.

Anaerobic granular sludge (AGS) primarily consists of bacteria and archaea. An examination of the architecture of AGS (Fig. 5) using FISH and confocal laser-scanning microscopy (CLSM) showed that the Bacteria domain (red, a1–e1) and Archaea domain (green, a2–e2) were the primary AGS components and consisted of the phyla α-Proteobacteria (red, a3–e3), β-Proteobacteria (rose, a4–e4), and Bacteroidetes (blue, a5–e5) as well as the genus *Methanosaeta* (green, a6–e6). Furthermore, either 500 mg/g TSS Ag NPs (b1–b6) or 500 mg/g TSS MgO NPs (c1–c6) caused a significant decrease in the center of AGS. Further quantification showed that 10 mg/g TSS nZVI (d1–d6) and 100 mg/g TSS Fe_2_O_3_ NPs (e1–e6) produced more active Bacteria (red, d1–e1), Archaea (green, d2–e2), α-Proteobacteria (red, d3–e3), β-Proteobacteria (rose, d4–e4), Bacteroidetes (blue, d5–e5) and *Methanosaeta* (green, d6–e6). α-Proteobacteria (red, a3–e3), β-Proteobacteria (rose, a4–e4), and Bacteroidetes (blue, a5–e5) are bacteria, which may affect hydrolysis and acidogenesis. The genus *Methanosaeta* (green, a6–e6) belongs to the Archaea domain, members of which play a key role in methanogenesis. With 500 mg/g TSS Ag NPs or 500 mg/g TSS MgO NPs, there were lower levels of active α-Proteobacteria (red, b3–c3), β-Proteobacteria (rose, b4–c4), and Bacteroidetes (blue, b5–c5); however, there were more active *Methanosaeta* (green, d6–e6) with 10 mg/g TSS nZVI or 100 mg/g TSS Fe_2_O_3_ NPs.

**Figure 6:**
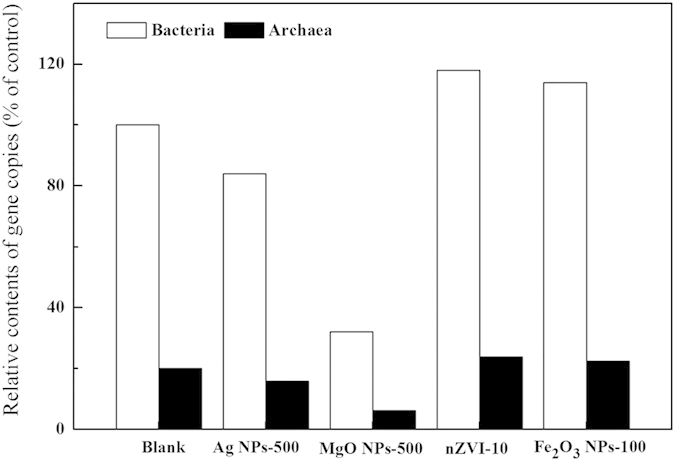
Fluorescent quantitative PCR results from five conditions (blank, Ag NPs-500, MgO NPs-500, nZVI-10 and Fe_2_O_3_ NPs-100).

To further describe the impacts of different dosages of Ag NPs, MgO NPs, nZVI and Fe_2_O_3_ NPs on the abundance of bacteria and archaea in an anaerobic digestion system, real-time FQ-PCR assays were used to determine the abundance of the microbial community after exposure to Ag NPs, MgO NPs, nZVI or Fe_2_O_3_ NPs. Typically, bacteria and archaea genes are widely used as functional markers of anaerobic digestion microorganisms. The quantitative changes in anaerobic digestion microorganisms can also typically be evaluated by measuring the numbers of copies of bacterial and archaeal genes^22^. The numbers of copies of bacterial and archaeal genes, respectively, increased to 118% and 114% of the control after exposure to 10 mg/g TSS nZVI and to 119% and 112% of the control after exposure to 100 mg/g TSS Fe_2_O_3_ NPs (Fig. 6).

**Figure 7:**
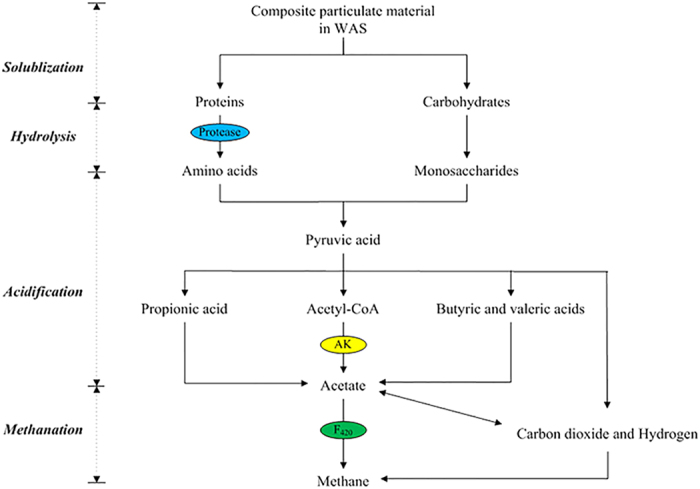
Proposed metabolic pathway for methane generation during WAS anaerobic digestion. Only the key enzymes that were assayed in this study are labeled.

**Figure 8:**
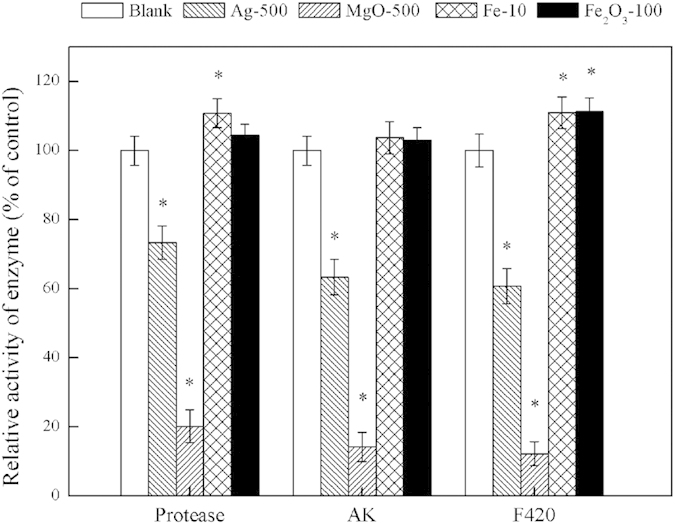
Comparisons of the activities of protease, AK and coenzyme F_420_ in the reactors exposed to 500 mg/g TSS Ag NPs, 500 mg/g TSS MgO NPs, 10 mg/g TSS nZVI or 100 mg/g TSS Fe_2_O_3_ NPs over the long term. Error bars represent standard deviations of triplicate tests.

Although many enzymes are related to the anaerobic digestion of sludge, only protease, AK and coenzyme F_420_ were detected as examples that were responsible for hydrolysis, acidification and methanation, respectively, in this study (Fig. 7). The relative activities of these enzymes in reactors over the long term are shown in Fig. 8 and Table S2 (Supplementary Information). The protease activity increased in the reactor exposed to 10 mg/g TSS nZVI over the long term but significantly decreased in the reactors exposed to 500 mg/g TSS Ag NPs and to 500 mg/g TSS MgO NPs over the long term. Ag NP and MgO NP dosages of 500 mg/g significantly decreased AK activity. nZVI and Fe_2_O_3_ NP dosages of 10 and 100 mg/g TSS, respectively, each increased the coenzyme F_420_ activities to 111% of the control, whereas the coenzyme F_420_ activities decreased to 60.73% and 12.26% of the control with Ag NPs and MgO NPs, respectively, each at 500 mg/g TSS.

## Discussion

Previous publications reported that the toxic metal ions released from the dissolution of NPs were primarily responsible for their toxicity to certain living organisms^19^,^23^,^24^. This mechanism could explain the toxicity of soluble NPs, such as ZnO NPs^21^. However, in the present study, enhancement of methane production was observed at a lower concentration of nZVI (i.e., 10 mg/g TSS), whereas methane production was inhibited by higher concentrations of Ag NPs and MgO NPs (i.e., 500 mg/g TSS). The literature has reported that SiO_2_ NPs induced no significant effects on human cells (e.g., MSTO and 3T3 cells) due to their insolubility, whereas ZnO NPs were strongly toxic due to their dissolution^23^. However, the present study found that methane production increased at lower concentrations of Fe_2_O_3_ NPs due to their insolubility, whereas a lower dosage of nZVI caused a significant increase in methane generation due to its dissolution. Although certain nanomaterials, such as Au, Ag and Fe_3_O_4_, have been reported to exert only marginal effects on anaerobic communities^25^,^26^, it is difficult to identify the different effects of NPs (i.e., nZVI, Ag NPs, Fe_2_O_3_ NPs and MgO NPs) on the microorganisms in WAS anaerobic digestion systems based on the effects of current NPs because multiple species of bacteria exist in a WAS digestion system. The impacts of NPs (e.g., nZVI, Ag NPs, Fe_2_O_3_ NPs and MgO NPs) on microorganism communities involved in anaerobic digestion systems have not been investigated to date; the present study addressed this topic in detail to describe the mechanisms of different NPs that affect the methane generated from the anaerobic digestion of sludge.

Researchers have found that many NPs can be adsorbed onto and/or reacted with cell membranes and can then disrupt them. Zhang *et al.* observed that ZnO NPs damaged the bacterial cell membrane^27^; Ma *et al.* believed that the disruption of a cell membrane and/or cell death was due to the physical penetration of CeO_2_ NPs and the oxidizing effect of dissolved Ce^4+^ on the outer membranes of microorganisms in an anaerobic digestion system^28^. However, other studies used LDH release assays to study the interactions of microbial cell membranes with various morphologies and dosages of NPs. The results of those assays indicated that the levels of lactate dehydrogenase, which is a marker of cell membrane damage, in the reactors exposed to 500 mg/g TSS Ag NPs or 500 mg/g TSS MgO NPs were considerably higher than those in the reactors exposed to 10 mg/g TSS nZVI or 100 mg/g TSS Fe_2_O_3_ NPs, suggesting that high concentrations of Ag NPs or MgO NPs could damage cell membranes.

Most researchers believe that the metal ions released from NPs play an important role in microorganism communities that are involved in biological treatment processes. Some researchers found that higher dosages of metal ions released from NPs inhibited these microorganism communities during the sewage sludge treatment process. Xia *et al.* demonstrated that the toxicity of ZnO NPs to the lysosomes and mitochondria of microorganism communities was due to the disruption of intracellular Zn homeostasis^19^. Other researchers found that the optimal concentration of released metallic ions could promote the activities of microbial communities. Li *et al.* showed that Fe^2+^ released from nZVI could combine with S^2−^ during the anaerobic digestion process to relieve the inhibitory effects of S^2−^ on microbial communities^20^. Zandvoort showed that Fe^2+^ was an important constituent in the assembly of iron-sulfur clusters and was responsible for the electron transport process in cellular redox activity^29^.

Direct interspecies electron transfer (DIET) via Fe_2_O_3_ NPs may play an important role in facilitating the methanogenesis process during the anaerobic digestion of sludge. In an anaerobic digestion system (e.g., anaerobic soils, sediments), fermentation bacteria can metabolize intermediate products, such as volatile fatty acids and ethanol, into acetate, carbon dioxide and reduced electron carriers. Traditionally, the reduced electron carriers are believed to regenerate into H_2_ or formate, which can serve as an electron donor for methane production by combining with CO_2_. This model for syntrophy and methanogen electron exchange is considered to be an interspecies electron transfer (IET)^30^,^31^. However, a potential and perhaps more efficient pathway of electron exchange, called DIET, was reported in the literature^32^,^33^. During this process, conductive materials that are either naturally occurring or artificially supplemented may be used for electron transfer. Fe_2_O_3_ NPs, which are a type of semi-conductive mineral, serve as electron conduits between the electron donors and acceptors and accelerate methane production from the reduced electron carriers and CO_2_^34^,^35^. The NPs in this process are similar to enzymes in catalytic reactions in a sequence of biochemical reactions^36^.

Previous studies reported that the Bacteria α-Proteobacteria, β-Proteobacteria and Bacteroidetes were related to the acidification process, whereas Archaea and *Methanosaeta* were responsible for the methanogenesis process^22^,^37^. The architecture of AGS showed that the Bacteria domain and Archaea domain were the primary AGS components, which consisted of α-Proteobacteria-phylum, β-Proteobacteria-phylum, Bacteroidetes-phylum, and *Methanosaeta*-genus. Further quantification showed that low concentrations of nZVI and Fe_2_O_3_ NPs (i.e., 10 mg/g TSS nZVI and 100 mg/g TSS Fe_2_O_3_ NPs) produced more active Bacteria, Archaea, α-Proteobacteria, β-Proteobacteria, Bacteroidetes and *Methanosaeta*. With higher concentrations of Ag NPs and MgO NPs (i.e., 500 mg/g TSS Ag NPs and 500 mg/g TSS MgO NPs), there were less active α-Proteobacteria, β-Proteobacteria, Bacteroidetes and *Methanosaeta*, which caused a significant decrease in the center of AGS. Obviously, exposure to different dosages of Ag NPs, MgO NPs, nZVI and Fe_2_O_3_ NPs caused a shift in the microbial community structure of the anaerobic digestion system. To further describe the impact of different dosages of Ag NPs, MgO NPs, nZVI and Fe_2_O_3_ NPs on the abundances of bacteria and archaea in an anaerobic digestion system, real-time fluorescence quantitative PCR was used to determine the abundance of the microbial community after exposure to Ag NPs, MgO NPs, nZVI and Fe_2_O_3_ NPs. The numbers of copies of bacterial and archaeal genes were decreased to 84% and 32% of the control, respectively, after exposure to 500 mg/g TSS Ag NPs and to 79% and 31% of the control, respectively, after exposure to 500 mg/g TSS MgO NPs. These results indicated that exposure to 500 mg/g TSS Ag NPs or 500 mg/g TSS MgO NPs decreased the abundance of anaerobic digestion microorganisms, whereas exposure to 10 mg/g TSS nZVI or 100 mg/g TSS Fe_2_O_3_ NPs increased the abundance of anaerobic digestion microorganisms, thus providing insights into the effects of different NPs on sludge digestion. Although many enzymes are related to the anaerobic digestion of sludge, only protease, AK and coenzyme F_420_ were identified as responsible for hydrolysis, acidification and methanation, respectively, in this study. These results corresponded with the experimental results regarding the effects of nanoparticle exposure on methane production. Different dosages of NPs produced different effects on the anaerobic digestion of sludge due to certain key enzymes.

## Methods

### NPs and Sludges

Fe NPs (nZVI) (<50 nm), Ag NPs (<100 nm), Fe_2_O_3_ NPs (<30 nm) and MgO NPs (<50 nm) were purchased from Sigma Aldrich (St. Louis, MO, USA). Suspensions of nZVI, Ag NPs, Fe_2_O_3_ NPs and MgO NPs stock (2000 mg/L) were prepared by adding NPs to distilled water (pH 7.0) containing sodium dodecylbenzene sulfonate (SDBS, 0.1 mM) to increase the dispersity of the NPs, followed by 1 h of ultrasonication (40 kHz, 250 W). Dynamic light scattering (DLS) using a Malvern Autosizer 4700 (Malvern Instruments, UK) device was conducted to measure the average size of the NPs (Ag NPs: 170 ± 7.9 nm, MgO NPs: 154 ± 7.9 nm, nZVI: 128 ± 7.9 nm, Fe_2_O_3 _NPs: 108 ± 7.9 nm). X-ray diffraction (XRD) analysis was conducted using a Rigaku D/Max-RB diffractometer that was equipped with a rotating anode and a Cu Ka radiation source, as shown in Fig. S2 (Supplementary Information). Transmission electron microscopy (TEM) images of the NPs were acquired from a Tecnai F20 (Philips Electron Optics, Netherlands) using an accelerating voltage of 200 kV to visualize their shape (Fig. S3, Supplementary Information).

The WAS used in this study was obtained from the secondary sedimentation tank of a municipal WWTP in Shanghai, China. After being concentrated via settling at 4 °C for 24 h, its primary characteristics (i.e., average ± standard deviations of triplicate tests) can be described as follows: pH = 6.4 ± 0.2; total suspended solids (TSS) = 21,840 ± 851 mg/L; volatile suspended solids (VSS) = 16,950 ± 695 mg/L; soluble chemical oxygen demand (SCOD) = 244 ± 8 mg/L; total chemical oxygen demand (TCOD) = 23,730 ± 1185 mg/L; total carbohydrate = 2,692 ± 113 mg-COD/L; and total protein = 13,443 ± 509 mg-COD/L. Anaerobic granular sludge (AGS), which was used as an inoculum, had been cultured in a laboratory upflow anaerobic sludge blanket (UASB) for 30 d with the addition of synthetic wastewater (Table S3, Supplementary Information), in which glucose (2,500 mg/L) was the primary carbon source. The UASB used in these experiments was made of Teflon and had a working volume of 4.5 L (diameter of 90 mm and height of 700 mm). The hydrolytic retention time of the UASB was 6 h, and the AGS concentration in the UASB was approximately 29,165 ± 1,225 mg/L. The characteristics of the WAS and AGS after settling are provided in Table S4 (Supplementary Information).

### Dissolution of NPs

To determine the dosage of the released metal ions from nZVI, Ag NPs, Fe_2_O_3_ NPs and MgO NPs, four dosages (i.e., 1, 10, 100 and 500 mg/g-TSS) of nZVI, Ag NPs, Fe_2_O_3_ NPs and MgO NPs in 0.1 mM SDBS solutions were prepared with the sludge. The mixtures were then placed in 16 identical reactors with a working volume of 1 L each at 35 ± 1 °C and 120 rpm for 2 days. The mixtures were withdrawn from the reactors and centrifuged at 12,000 g for 30 min, creating a supernatant, which was then filtered through a 0.22 μm mixed cellulose ester membrane. After acidification with 4% ultrahigh-purity HNO_3_, the released metal ions were detected via inductively coupled plasma optical emission spectrometry (ICP-OES, PerkinElmer Optima 2100 DV, USA). In this study, among the four NPs investigated, nZVI, Ag NPs and MgO NPs showed significant metal ion (Fe^2+^, Ag^+^ and Mg^2+^) release; the release of metal ions was negligible in the suspension of Fe_2_O_3_ NPs.

### Experiments on the Effects of NPs and their Released Metal Ions on the WAS Anaerobic Digestion for Methane Production

To investigate the effects of nZVI, Ag NPs, Fe_2_O_3_ NPs and MgO NPs on WAS anaerobic digestion, the concentrations of nZVI, Ag NPs, Fe_2_O_3_ NPs and MgO NPs were set at 1, 10, 100, and 500 mg/g TSS, respectively, based on the environmentally relevant concentrations and possible future risks caused by large-scale nanoparticle applications. In the literature, a concentration of 1 mg/g TSS NPs was considered to be an environmentally relevant dosage; however, the potential impacts of higher dosages of NPs were also studied because the environmental release of NPs might be increased due to large-scale production^21^. Meanwhile it can provide basis and polluting fore-warning for anaerobic digestion of sludge from factories in which NPs were produced or used. The effects of exposure to nZVI, Ag NPs, Fe_2_O_3_ NPs and MgO NPs on methane production were investigated in 18 identical reactors with a working volume of 1 L each at 35 ± 1 °C and 120 rpm for 30 days. Raw sludge with a volume of 8.46 L was divided equally into these 18 reactors. Then, 30 mL of anaerobic granular sludge was added to each reactor to produce methane. Because SDBS was used as the dispersing reagent, two controls (reactors 1–2; one with only sludge, and one with sludge and SDBS (0.1 mM)) were performed to determine whether methane production was influenced by the addition of SDBS. The nZVI, Ag NPs, Fe_2_O_3_ NPs and MgO NPs were added into the other 16 reactors^3^,^4^,^5^,^6^,^7^,^8^,^9^,^10^,^11^,^12^,^13^,^14^,^15^,^16^,^17^,^18^ at dosages of 1, 10, 100 and 500 mg/g TSS, respectively. The total gas volume was measured daily based on a previous publication^22^. Stock solutions (50 mg/L) of FeCl_2_, AgNO_3_ and MgCl_2_ were combined with 0.1 mM SDBS. The experiments on the effects of the released metal ions from the NPs were also performed using the method described above but with FeCl_2_, AgNO_3_ and MgCl_2 _instead of the nZVI, Ag NPs and MgO NPs. The dosages of Fe^2+^, Ag^+^ and Mg^2+^ added to the reactors were 1.3, 4.6, 9.3, 0.52, 0.8, 3.3, 0.2, 1.2, 4.8, and 9.8 mg/L.

### Effects of NPs on the Anaerobic Granular Sludge Surface and Microorganism Community during Long-Term Reactor Operation

Five semi-continuous-flow reactors with a working volume of 1.0 L each were operated. The five reactors each received 470 mL of raw sludge, which was cultured over the long term in the absence of NPs and in the presence of 500 mg/g TSS Ag NPs, 500 mg/g TSS MgO NPs, 10 mg/g TSS nZVI and 100 mg/g TSS Fe_2_O_3_ NPs. Every day, 15 mL of the fermentation mixture was manually withdrawn from each reactor, and the same amounts of sludge and NPs were added to each reactor. After operating for nearly 3 months, the methane production remained stable; at this point, the anaerobic granular sludge surface, microbial community and enzyme activities were analyzed.

### Other Analytical Methods

TSS, VSS, TCOD, SCOD, carbohydrate and protein were analyzed based on a previous publication^22^. The released metal ions were detected using inductively coupled plasma optical emission spectrometry (ICP-OES, PerkinElmer Optima 2100 DV, USA). Details regarding protease activity, acetate kinase (AK) activity, coenzyme F_420_ activity, lactate dehydrogenase (LDH) release, and scanning electron microscopy (SEM) analyses are provided in the Supplementary Information.

FISH with 16S rRNA-targeted oligonucleotide probes was used to intuitively detect changes in microbial distribution in the AGS used as the inoculums when the sludge was exposed to different NPs during the anaerobic digestion process. The fixation and sectioning of AGS were conducted as follows. Samples from the reactors were fixed in freshly prepared 4% paraformaldehyde solution for 4 h at 4 °C before being eluted three times with 1× phosphate-buffered saline (PBS). The solution was then suspended in 1:1 1× PBS and 100% ice-cold ethanol (vol/vol). These samples were then dehydrated by successively passaging through 50%, 80%, and 100% ethanol three times; 1:1 (vol/vol) ethanol-xylene; and 100% xylene three times. The resulting material was then embedded in melted paraffin wax. Serial sections that were 10–15 μm long were cut with a rotary microtome and mounted on gelatin-coated glass slides. The sections were dewaxed using 100% xylene twice and 100% ethanol twice. They were then dried at room temperature for the FISH experiments. The following 16S rRNA-targeted oligonucleotide probes were used: Bacteria hybridized with ALF968, labeled with CY-3; Archaea hybridized with ARC915, labeled with FAM; α-Proteobacteria hybridized with ALF968, labeled with CY-3; β-Proteobacteria hybridized with BET42a, labeled with CY-5; Bacteroidetes hybridized with CFB719, labeled with HEX; and *Methanosaeta* hybridized with MX825, labeled with FAM. These 16S rRNA-targeted oligonucleotide probes are described in detail in Table S5 (Supplementary Information). Hybridizations were performed in a hybridization incubator (ThermoBrite, USA) with a buffer containing 0.9 M NaCl, 20 mM Tris-HCl, 0.01% sodium dodecyl sulfate and 5 ng of each labeled probe/mL at 46 °C for 2.5 h. The wash step was conducted at 48 °C for 30 min with wash buffer (0.9 M NaCl, 20 mM Tris-HCl, 0.01% sodium dodecyl sulfate). Then, the sections hybridized with the probes were observed with a confocal laser-scanning microscope (CLSM, Leica TCS, SP2 AOBS).

Real-time FQ-PCR was performed to determine the absolute genetic content of bacteria (P338F, P518R) and archaea (ARC109F, ARC344R) (Table S6, Supplementary Information). The analytical procedures are detailed in the Supplementary Information.

### Statistical Analysis

All tests were performed in triplicate, and the significance of the results was determined using analyses of variance (ANOVAs). *p* < 0.05 was considered to be statistically significant.

## Additional Information

**How to cite this article**: Wang, T. *et al.* Effects of Metal Nanoparticles on Methane Production from Waste-Activated Sludge and Microorganism Community Shift in Anaerobic Granular Sludge. *Sci. Rep.* **6**, 25857; doi: 10.1038/srep25857 (2016).

## Supplementary Material

Supplementary Information

## Figures and Tables

**Figure 1:**
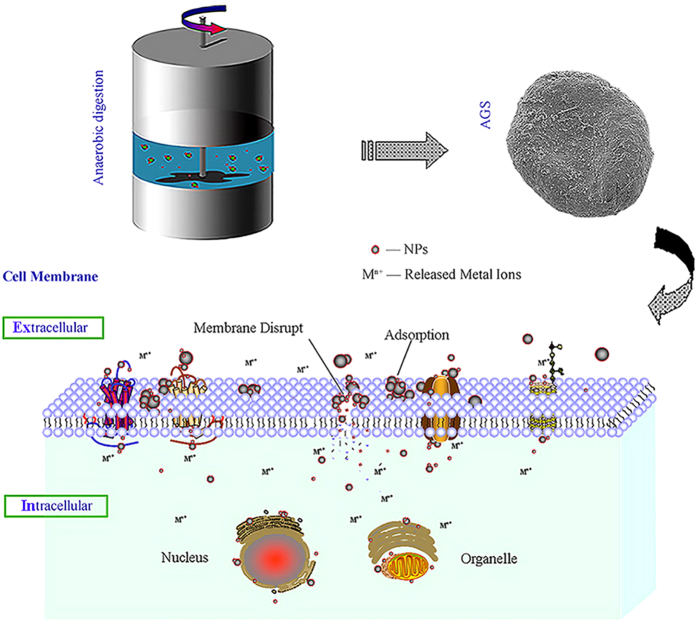
Effects of metal nanoparticles on an anaerobic digestion system.
