# Supplementary material for: Effects of Metal Nanoparticles on Methane Production from Waste-Activated Sludge and Microorganism Community Shift in Anaerobic Granular Sludge
Source: Sci Rep. 2016 May 11;6:25857. doi: 10.1038/srep25857 (PMC4863170; doi:10.1038/srep25857)
Supplement: Supplementary Information [file srep25857-s1.pdf]

Supplementary Information

Effects of Metal Nanoparticles on Methane Production from Waste-Activated Sludge and Microorganism Community Shift in Anaerobic Granular Sludge

Tao Wang, Dong Zhang*, Lingling Dai, Yinguang Chen, Xiaohu Dai*

(State Key Laboratory of Pollution Control and Resources Reuse, School of Environmental Science and Engineering, Tongji University, 1239 Siping Road, Shanghai 200092, China)

*Corresponding author

Dong Zhang Phone: 86-21-55126332, Fax: 86-21-65983602, E-mail: zhangdong_2011@aliyun.com

Xiaohu Dai Phone: 86-21-65983868, Fax: 86-21-65986313, E-mail: daixiaohu@tongji.edu.cn

Supporting Information: 8 pages, 6 tables, 3 figures

Determination of the Activities of Protease, AK and Coenzyme F_{420}.

Protease activity was determined based on the methods of Goel et al. (*1*) using ρ-nitrophenyl phosphate disodium salt as the standard. To determine the AK activity, 25 mL of the digestion mixture was obtained from the anaerobic reactors and then washed. The mixture was resuspended in 10 mL of 100 mM sodium phosphate buffer (pH 7.4) and then sonicated at 20 kHz for 30 min to break down the bacterial cells. Then, the substrates were centrifuged at 10,000 rpm at 4°C for 30 min to remove the waste debris; the extracts were kept on ice for the AK activity assay. AK activity was analyzed based on the methods of Allen at al. (*2*). The activity of coenzyme F_{420} was measured using the ultraviolet spectrophotometry method (*3*). The enzyme activities of protease and coenzyme F_{420} are described as units of enzyme activity per milligram of VSS (units/mg-VSS), and the activity of AK is described as units of enzyme activity per milligram of protein (units/mg-protein).

Scanning Electron Microscopy (SEM).

SEM was used to characterize the surface morphology of the activated sludge. Twenty milliliters of the mixture described above was withdrawn from the reactors and centrifuged at 3,000 rpm for 10 min. After being washed three times with 0.1 M phosphate buffer (pH 7.4), the centrifuged pellets were fixed in 0.1 M phosphate buffer (pH 7.4) containing 2.5% glutaraldehyde for 4 h at 4°C. The pellets were washed three times with 0.1 M phosphate buffer; dehydrated in 50%, 70%, 90% and 100% ethanol solutions for 15 min each; and then air-dried.

Lactate Dehydrogenase (LDH) Release Assays.

Lactate dehydrogenase release assays were conducted to measure the cell membrane integrity in relation to exposure to the NPs using a cytotoxicity detection kit (Roche Applied Science) according to the manufacturer's instructions. After operation for nearly 3 months, the mixture was centrifuged at 12,000 g for 5 min, and the cell-free culture supernatant was seeded on a 96-well plate. Then, 50 μL of reagent mixture was added to each sample and incubated at room temperature in the dark. After 30 min of incubation, 50 μL of stop solution was added to each well. Finally, the absorbance was determined using a microplate reader (BioTek, USA) at a wavelength of 490 nm.

Quantitative Real-time Polymerase Chain Reaction (PCR).

Real-time PCR was used to quantify the Bacteria and Archaea (methanogens) present to create functional markers. The primers P338F-(5'-ACTCCTACGGGAGGCAG-3') and P518R-(5'-ATTACCGCGGCTGCTGG-3') and the primers ARC109F-(5'-ACKGCTCAGTAACACGT-3') and ARC344R-(5'-TCGCGCCTGCTGCTCCCCGT-3') were used to amplify the Bacterial and Archaeal gene fragments, respectively (*4*, 5). PCR was performed in a total volume of 20 μL containing 1× SYBR Green PCR Master Mix (Invitrogen), primers for Bacteria or Archaea (0.5 μM each), and 1 μL of template DNA. A standard curve was generated using 10 serial dilutions of linearized plasmids that contained the cloned Bacterial or Archaeal gene as a template. This curve was then used for the absolute quantification of Bacterial or Archaeal gene copies. PCR was performed using three replicates per sample and included control reactions without a template.

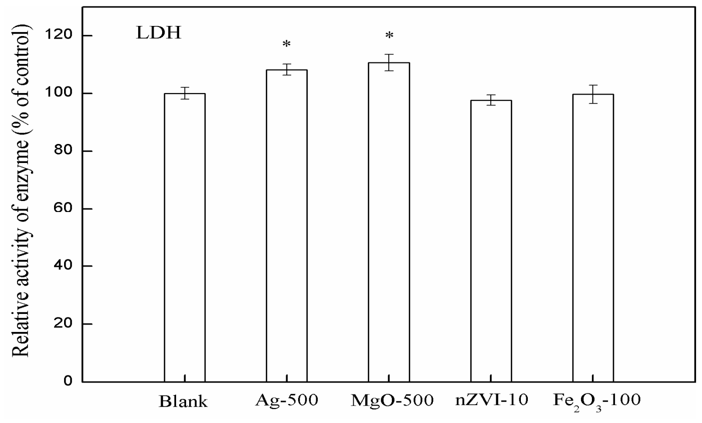

Figure S1. Comparisons of the activities of LDH in the long-term-operated reactors exposed to 500 mg/g TSS Ag NPs, 500 mg/g TSS MgO NPs, 10 mg/g TSS nZVI or 100 mg/g TSS Fe_2O_3 NPs. Error bars represent standard deviations of triplicate tests.

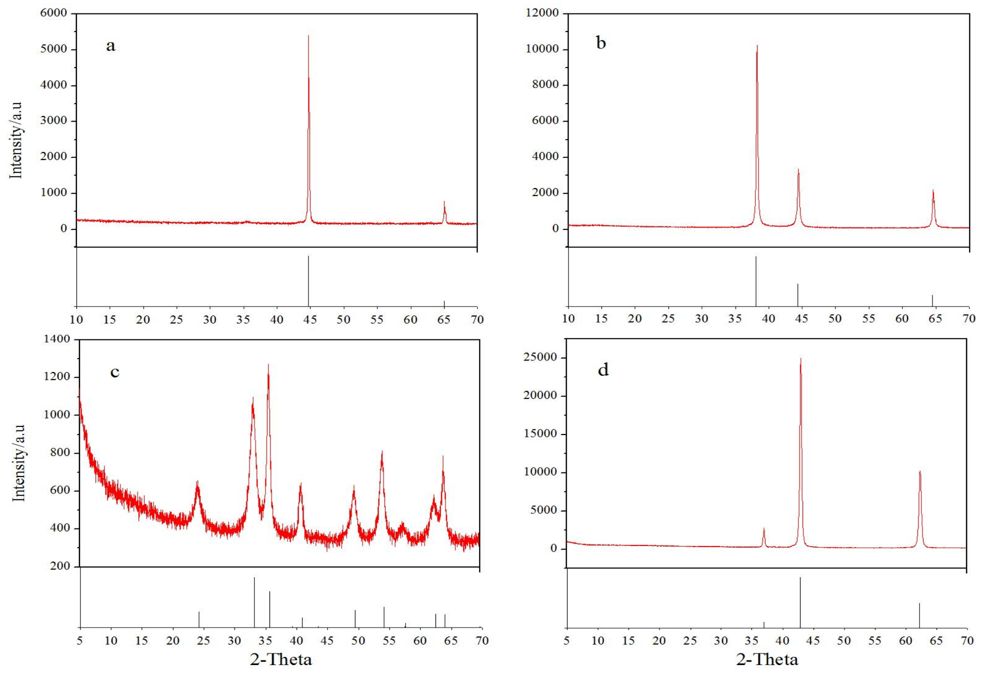

Figure S2. XRD patterns of nanoparticle powders: nZVI (a), Ag NPs (b), Fe_2O_3 NPs (c) and MgO NPs (d).

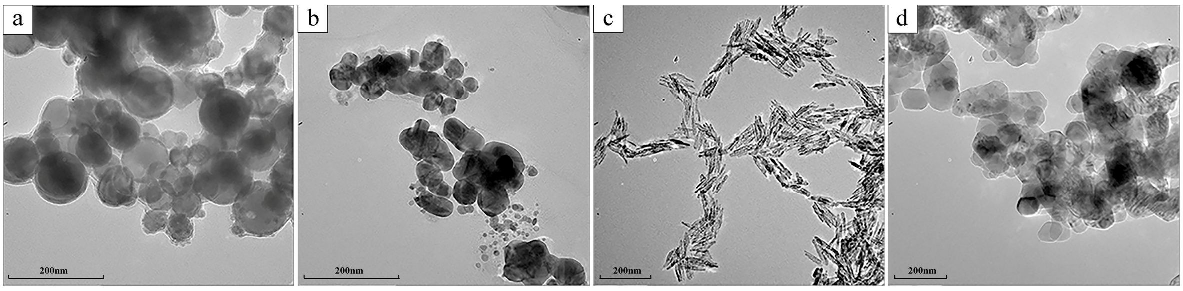

Figure S3. Transmission electron microscopy (TEM) micrographs of nanoparticles: nZVI (a), Ag NPs (b), Fe_2O_3 NPs (c) and MgO NPs (d).

Table S1. Statistical Analyses of Different Dosages of NPs and Their Released Ions Affecting Methane Production Compared to the Control.

	(mg/g TSS)	$F_{observed}$	$F_{significance}$	$P_{0.05}$		(mg/L)	$F_{observed}$	$F_{significance}$	$P_{0.05}$
nZVI	1	0.80	7.71	0.42	Fe^{2+}	ND	—	7.71	—
	10	58.23	7.71	1.58×10^{-3}		1.3	27.25	7.71	6.43×10^{-3}
	100	25.68	7.71	7.14×10^{-3}		4.6	11.21	7.71	0.03
	500	5.49	7.71	0.08		9.3	2.85	7.71	0.17
Ag NPs	1	0.68	7.71	0.46	Ag^{+}	ND	—	7.71	—
	10	3.16	7.71	0.15		5.2×10^{-2}	5.91×10^{-3}	7.71	0.94
	100	0.97	7.71	0.38		0.8	0.73	7.71	0.44
	500	147.36	7.71	2.64×10^{-4}		3.3	125.34	7.71	3.62×10^{-4}
Fe_2O_3 NPs	1	2.11	7.71	0.22	Fe^{3+}	ND	—	7.71	—
	10	5.45	7.71	0.08		ND	—	7.71	—
	100	46.17	7.71	2.45×10^{-3}		ND	—	7.71	—
	500	50.31	7.71	0.08		ND	—	7.71	—
MgO NPs	1	0.35	7.71	0.58	Mg^{2+}	0.16	2.32	7.71	0.20
	10	0.57	7.71	0.49		1.24	0.12	7.71	0.75
	100	2045.70	7.71	1.43×10^{-6}		4.81	2.81	7.71	4.71×10^{-3}
	500	3546.10	7.71	4.76×10^{-7}		9.81	123.05	7.71	3.76×10^{-4}

Table S2. Statistical Analyses of Key Enzyme Activities Affected by Different Concentrations of NPs.

		$F_{observed}$	$F_{significance}$	$P_{0.05}$			$F_{observed}$	$F_{significance}$	$P_{0.05}$
Protease	Ag-500	51.44	7.71	2.00×10^{-3}	AK	Ag-500	92.17	7.71	6.58×10^{-4}
	MgO-500	477.21	7.71	2.60×10^{-5}		MgO-500	622.24	7.71	1.53×10^{-5}
	Fe-10	10.06	7.71	3.38×10^{-2}		Fe-10	1.06	7.71	0.36
	Fe_2O_3-100	2.01	7.71	0.23		Fe_2O_3-100	0.94	7.71	0.39
F_{420}	Ag-500	94.94	7.71	6.21×10^{-4}	LDH	Ag-500	25.79	7.71	7.09×10^{-3}
	MgO-500	645.63	7.71	1.42×10^{-5}		MgO-500	38.00	7.71	3.51×10^{-3}
	Fe-10	8.08	7.71	4.67×10^{-2}		Fe-10	2.20	7.71	0.21
	Fe_2O_3-100	9.78	7.71	3.52×10^{-2}		Fe_2O_3-100	0.02	7.71	0.90

Table S3. Synthetic Wastewater Composition [a].

**Components**	**Concentration**	**Components**	**Concentration**
NH_4Cl	1000	H_3BO_3	0.5
KH_2PO_4	500	$(NH_4)_6Mo_7O_{24}\cdot4H_2O$	0.5
$CaCl_2$	200	$CoCl_2\cdot6H_2O$	0.5
$MgCl_2\cdot6H_2O$	200	$AlCl_3\cdot6H_2O$	0.5
$FeCl_3$	50	EDTA	4
$ZnSO_4\cdot7H_2O$	0.5	$MnCl_2\cdot4H_2O$	1
$CuSO_4\cdot5H_2O$	0.5	$NiCl_2\cdot6H_2O$	1

[a] Units: mg/L of tap water. Glucose (2,500 mg/L) was the primary carbon source.

Table S4. Characteristics of the Waste-Activated Sludge and Anaerobic Granular Sludge after Settling [a].

Parameter	WAS	AGS
pH	6.4 ± 0.2	7.0 ± 0.1
TSS (total suspended solids) [b]	21840 ± 851	32875 ± 1532
VSS (volatile suspended solids)	16950 ± 695	24732 ± 758
SCOD	244 ± 8	287 ± 11
TCOD	23730 ± 1185	35119 ± 1321
Total carbohydrate (as COD)	2692 ± 113	5340 ± 240
Total protein (as COD)	13443 ± 509	19945 ± 442

[a] Total carbohydrate and total protein are expressed in mg COD/L. The data shown are the averages and their standard deviations in duplicate tests.

[b] TSS, VSS, SCOD and TCOD are expressed in mg/L.

Table S5. FISH Oligonucleotide Probes Used in This Study (*6-8*).

Probe	Sequence (5'–3')	Specificity	Dye	Wavelength
EUB338	GCTGCCTCCCGTAGGAGT	Bacteria	CY-3	554-568
ARC915	GTGCTCCCCCGCCAATTCCT	Archaea	FITC	490-525
ALF968	GGTAAGGTTCTGCGCGTT	α-Proteobacteria	CY-3	554-568
BET42a	GCCTTCCCACTTCGTTT	β-Proteobacteria	CY-5	649-666
CFB719	AGCTGCCTTCGCAATCGG	Bacteroidetes	HEX	535-553
MX825	TCGCACCGTGGCCGACACCTAG	*Methanosaeta*	FITC	490-525

Table S6. Real-time Quantitative PCR Primers Used in This Study (*4*, *5*).

Probe	Gene	Sequence (5'–3')	Amplified fragment length
Bacteria	P338F	ACTCCTACGGGAGGCAG	199 bp
	P518R	ATTACCGCGGCTGCTGG	
Archaea	ARC109F	ACKGCTCAGTAACACGT	259 bp
	ARC344R	TCGCGCCTGCTGCTCCCCGT	

References

1. Goel, R., Mino, T., Satoh, H. & Matsuo, T. Enzyme activities under anaerobicandaerobic conditions in activated sludge sequencing batch reactor. *Water Res.* **32,** 2081–2088 (1998).
2. Allen, S., Kellermeyer, R., Stjernholm, R. & Wood, H. Purification and properties of enzymes involved in the propionic acid fermentation. *J. Bacteriol.* **87,** 171–187 (1964).
3. Delafontaine, M. J., Naveau, H. P. & Nyns, E. J. Fluorimetric monitoring of methanogenesis in anaerobic digesters. *Biotechnol. Lett.* **1,** 71–73 (1979).
4. Ovreås, L., Forney, L., Daae, F. L. & Torsvik, V. Distribution of bacterioplankton in meromictic Lake Saelenvannet, as determined by denaturing gradient gel electrophoresis of PCR-amplified gene fragments coding for 16S rRNA. *Appl. Environ. Microb.* **63,** 3367–3373 (1997).
5. Großkopf, R., Janssen, P. H. & Liesack, W. Diversity and structure of the methanogenic community in anoxic rice paddy soil microcosms as examined by cultivation and direct 16S rRNA gene sequence retrieval. *Appl. Environ. Microb.* **64,** 960–969 (1998).
6. Amann, R. I. *et al.* Combination of 16S rRNA-targeted oligonucleotide probes with flow cytometry for analyzing mixed microbial populations. *Appl. Environ. Microb.* **56,** 1919–1925 (1990).
7. Stahl, D. A., Flesher, B., Mansfield, H. R. & Montgomery, L. Use of phylogenetically based hybridization probes for studies of ruminal microbial ecology. *Appl. Environ. Microb.* **54,** 1079–1084 (1988).
8. Raskin, L., Stromley, J. M., Rittmann, B. E. & Stahl, D. A. Group-specific 16S rRNA hybridization probes to describe natural communities of methanogens. *Appl. Environ. Microb.* **60,** 1232–1240 (1994).
